# A multi-specialty surgical course for residents transitioning from early to intermediate training

**DOI:** 10.5116/ijme.5708.e9ea

**Published:** 2016-05-01

**Authors:** Daniel Glassman, Marina Yiasemidou, Balachandran Venkateswaran, Rangasamy Sivakumar, Sanjib Majumder, Chandra S. Biyani

**Affiliations:** 1 School of Surgery, Health Education Yorkshire and the Humber, Leeds, UK; 2 Mid Yorkshire Hospitals NHS Trust, Wakefield, West Yorkshire, UK; 3 St James's University Hospital, Leeds, UK

**Keywords:** Multi-specialty, surgical course, residents, intermediate training

## Introduction

It has been estimated that approximately 15000 to 20000 hours are required to train a surgeon.^1^ However, several time-constrains imposed globally^2^ have significantly reduced training time in the Operating Room (OR) posing a significant challenge for surgical educators and creating an impetus for the development of new educational methods.^3^ Since August 2005, all medical graduates in the United Kingdom must enter a 2 year postgraduate programme prior to commencing training in their chosen medical or surgical specialty.^4^

### What is the problem?

Despite this two-year programme being a good introduction to duties as a doctor there have been some concerns that the trainees do not gain significant experience in surgical procedures leading to reduced confidence.^5^ Which can make transition from a second year postgraduate (Foundation) doctor (PGY2) to a surgical resident particular difficult. To gain more experience a large number of doctors undertaking additional posts, which are not part of the recognised training scheme, in the UK and abroad, or roles such as anatomy demonstrators, in order to broaden their skills and relevant knowledge prior to progressing to surgical training.^6^

### What have we done?

We propose a multi-specialty surgical, simulation embedded course, designed for and addressed strictly to PGY2 doctors due to commence their surgical training in the UK. The course comprised of procedures and skills in general surgery, urology, trauma and orthopaedics and plastic surgery. The curriculum was developed by teams of experts, based on their clinical experience of what a junior surgical resident will be asked to do during the first year of their core surgical training and the national surgical training curriculum (ISCP).^7^

We used pretest-posttest design to assess improvement in (a) knowledge through multiple choice question test and (b) confidence level in procedures. The participants were twenty-three PGY2 doctors, fourteen males, nine females, who were scheduled to commence surgical residency within 3 months from the course. Participants were divided in to 4 groups and the course director assigned 6 participants to each group (urology, general surgery, plastic surgery and orthopaedics). Skills included urology (suprapubic catheterisation, flexible cystoscopy, circumcision [synthetic models] and scrotal exploration on animal models), plastic surgery (burns management, skin closure and hand fracture fixation [synthetic model], Orthopaedics (Thomas splint application, knee injection and aspiration [synthetic model], plastering, plating long bone fractures[synthetic models] and reducing shoulder dislocation) and general surgery (hand sewn bowel anastomosis, ileostomy, serosal tear repair and laparoscopic appendicectomy [animal model]). Each session was 4 hours long and included an introduction, followed by essential procedures in the specialty with debriefing. All participants rotated through each specialty in 2 days.  Four months after the completion of the course the confidence levels  for the same procedures were assessed in the same way.

Nineteen residents of similar training level, who did not participate in the course, were randomly selected and asked to score their confidence, using a similar questionnaire. The results from participants and non-participants were compared with each other in order to assess for medium term retainement of the immediate impact of the course.

### What lessons were learned?

Confidence in performing all the taught surgical skills was significantly improved after the completion of the course between participants and non-participants of the same training level four months after the completion of the course. Multiple Choice Question scores were also significantly improved after the completion of the course.

## Discussion

Although the effect of single specialty courses involving medical students, on confidence and knowledge are well documented^8^^-^^10^ however, there is a paucity of such evidence for post-graduate training. The current study demonstrates that a multi-specialty surgical course is feasible. It is also indicative that such a course can increase participants’ confidence in performing surgical procedures as well as related practical knowledge. Furthermore, it is likely that participants retain confidence for several months after the course and are more self-assured when compared to their peers who have not undertaken a surgical “hands on” course.  The results of the current study are in accordance with similar studies, single specialties performed both with the participation of pre-graduates^8^^-^^10^ and post-graduate^11^^,^^12^ doctors. Previous studies used similar methodology as our study with pre and post simulation assessments, in their vast majority utilising a questionnaire/survey as the assessment tool (all but cardiac surgery).^8^^, ^^9^^, ^^11^  

Despite this course being conducted to facilitate doctors commencing their surgical training in the UK, it may be relevant for other healthcare systems as time constrictions and reducd^2^^,^^9^ training opportunities are imposed in several countries. Furthermore, public intolerance to error (Intolerance of error and culture of blame drive medical excess) and drive to increase patient-safety, makes the employment of simulation training in surgery imperative.^13^ These are concepts that apply globally, making this course relevant outside the UK. Existing courses for surgical trainees in the early stages of their training are somewhat less inclusive of surgical sub-specialties, however not to the degree of the course described here which is equally inclusive of four surgical sub-specialties. Additionally, the course ventures beyond basic surgical skills and teaches full surgical procedures.

## Conclusions

A multi-specialty surgical course for intermediate experience residents is feasible and enhances residents’ confidence and theoretical knowledge. Transitioning from early years of postgraduate training to a surgical career in the United Kingdom (UK) can be facilitated with similar simulation embedded, “hands-on” courses. 

### Conflicts of Interest

The authors declare that they have no conflict of interest.
